# Overcoming Shifting Baselines: Paleo‐Behaviour Reveals Industrial Revolution as Tipping Point

**DOI:** 10.1111/gcb.70038

**Published:** 2025-01-25

**Authors:** Julian Lilkendey, Jens Hegg, Matthew Campbell, Jingjing Zhang, Harrison Raby, Malcolm Reid, Monica Tromp, Emma Ash, Louise Furey, Lindsey White, Richard Walter, Armagan Sabetian

**Affiliations:** ^1^ School of Science Auckland University of Technology Auckland New Zealand; ^2^ Department of Biology Gonzaga University Spokane Washington USA; ^3^ Anthropology Department University of Auckland Auckland New Zealand; ^4^ CFG Heritage Ltd. Auckland New Zealand; ^5^ The New Zealand Institute for Plant and Food Research Limited Auckland New Zealand; ^6^ Centre for Trace Element Analysis, Department of Geology University of Otago Dunedin New Zealand; ^7^ Department of Geology University of Otago Dunedin New Zealand; ^8^ Southern Pacific Archaeological Research, School of Social Sciences University of Otago Dunedin New Zealand; ^9^ Auckland War Memorial Museum Tāmaki Paenga Hira Auckland New Zealand; ^10^ Auckland New Zealand; ^11^ School of Social Sciences University of Queensland Brisbane Australia

**Keywords:** global change, habitat restoration, industrialisation, land‐use change, otolith microchemistry, Palaeoecology, time series analysis

## Abstract

Human activities have significantly altered coastal ecosystems worldwide. The phenomenon of shifting baselines syndrome (SBS) complicates our understanding of these changes, masking the true scale of human impacts. This study investigates the long‐term ecological effects of anthropogenic activities on New Zealand's coastal ecosystems over 800 years using fish otolith microchemical profiling and dynamic time warping across an entire stock unit. Results reveal a shift in snapper ( *Chrysophrys auratus* ; Sparidae) habitat‐use behaviour, transitioning from low‐salinity estuarine environments to higher‐salinity habitats, correlating with ongoing land‐use changes. This shift coincided with New Zealand's localised Industrial Revolution, which served as a tipping point for widespread ecosystem transformation. By comparing current coastal fish movement profiles with historical baselines, we provide evidence to address SBS and guide conservation strategies. Re‐establishing pre‐industrial habitat‐use behaviours in snapper will indicate successful habitat restoration, promoting overall ecosystem connectivity and resilience. Our findings enable more effective habitat restoration measures and sustainable management practices, informing policies for maintaining coastal biodiversity and ecosystem function.

## Graphical abstract

Lilkendey et al. explore how anthropogenic pressures, including industrialisation over the past 800 years, have altered the habitats and behaviours of New Zealand snapper. Using chemical analysis of snapper otoliths (ear stones) from archaeological, historical, and modern samples, the study reveals a shift in habitat use, from low‐salinity estuarine nurseries to higher‐salinity marine environments. The research highlights the impact of shifting baselines syndrome, where gradual environmental degradation resets perceptions of ecosystem health, masking the true extent of habitat loss. Their findings offer critical insights for conservation and habitat restoration efforts, underscoring the importance of addressing long‐term environmental changes in ecosystem management.

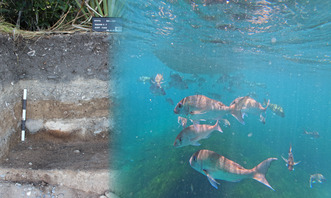

## 1 Introduction

The industrial age, marked by rapid technological advancements and urban expansion, has exerted immense pressure on ecosystems globally (Steinberg 1986). Particularly, coastal regions are characterised by dense human populations living alongside diverse habitats that support a myriad of marine organisms. These regions, which provide crucial ecosystem services for coastal communities, face challenges exacerbated by global change (Lu et al. 2018). The cumulative effects of increased industrialisation and agribusiness are likely to intensify eutrophication in coastal waters, resulting in reduced water quality and loss of habitat (Rabalais et al. 2009). A key obstacle in addressing these issues is the shifting baselines syndrome (SBS), a concept pivotal for understanding long‐term ecological changes. SBS highlights how generational shifts in environmental perception often overlook historical baselines, leading to a significant underestimation of human‐induced ecological changes in coastal ecosystems (Papworth et al. 2009).

Since the Industrial Revolution, industrialisation has significantly reshaped global ecosystems, with its wide‐ranging effects contributing to climate change, rising sea levels, overexploitation of biological resources, and the degradation of coastal habitats (Watson et al. 2023). This pervasive influence of anthropogenic activities has made the understanding of historical baselines crucial to addressing the inherent biases in ecological findings. Recent research highlights significant changes in biodiversity within terrestrial and marine ecosystems driven by human activities (Blowes et al. 2019). For example, in the Caribbean the decline in coral cover over decades, exacerbated by industrial stressors, has led to a marked shift in perceptions of coral reef health among successive generations of experts (Muldrow, Parsons, and Jonas 2020). Notably in New Zealand, Lyver et al. (2021) documented intergenerational differences in the perception of species abundance among inland Māori communities, illustrating SBS. Elders recalled significantly higher historic populations of New Zealand pigeon (kererū, *Hemiphaga novaeseelandiae* ), long‐finned eel (tuna, *Anguilla dieffenbachii* ), and North Island brown kiwi ( *Apteryx mantelli* ), while younger members considered today's reduced abundance of these species as normal. SBS presents a formidable challenge particularly in fisheries management, where the lack of high‐resolution historical data can lead to significant misinterpretations of current ecological conditions (Myers and Worm 2003). This is further compounded in the context of industrialised fishing, where inaccurate baselines can severely undermine the understanding and management of fish populations and habitats, leading to suboptimal conservation and management strategies (Pauly 1995). Addressing these challenges requires a concerted effort to integrate historical data with current environmental assessments, thereby enabling more effective responses to the complex and evolving challenges posed by industrialisation and its multifaceted impacts on marine ecosystems.

Fish have historically been recognised as excellent indicator organisms of coastal ecosystem health and function, owing to their high sensitivity to global changes (Candolin and Rahman 2023). The prevailing issues evoked by SBS can be alleviated through the use of time‐agnostic time‐series analysis techniques in sclerochronology, which allow for detailed profiling of the environmental history hard‐coded in fish otoliths, providing insights into past environmental conditions and fish behaviours (Campana and Thorrold 2001; Hegg and Kennedy 2021; Sabetian et al. 2021). Such a strategy is particularly effective in reconstructing paleoenvironments, enabling us to accurately determine ecological changes over extended periods (Disspain, Wallis, and Gillanders 2011). This methodology can overcome the limitations posed by SBS, providing a critical historical context for our current ecological assessments (Thomas and Swearer 2019) and helping to develop more targeted conservation strategies (Palumbi et al. 2003). Addressing the impacts of industrialisation and overcoming SBS is crucial not only for preserving marine biodiversity but also for ensuring the sustainability of marine resources for future generations, fostering an informed and effective approach to environmental stewardship in an era marked by rapid global change and increasing human impacts on ecosystems (Jackson et al. 2001; Pauly 1995).

Our study explores the complicated interplay between ecological dynamics and the scarcity of historical knowledge, applying a comprehensive approach that combines contemporary insights with empirical archaeological and historical data. We endeavour to assess the impacts of human activities on ecosystem health using a fish species as a sentinel, against the backdrop of global environmental change and the pressures it places on coastal ecosystems. Landscape‐scale changes, such as forest loss, have been shown to catalyse significant shifts in population dynamics and biodiversity, emphasising the complex biotic consequences of land‐use change (Daskalova et al. 2020). Focusing on the Auckland East (SNA1) stock of New Zealand snapper (tāmure, *Chrysophrys auratus* ), our research spans eight centuries to explore how anthropogenic activities have influenced their movement patterns, using these as an indicator for ecosystem health and contrasting modern‐day data with those from the pre‐industrial era. Past findings suggest that current assessments of snapper populations and the condition of their nursery habitats may reflect a baseline already skewed by human intervention, rather than an untouched, pre‐industrial state (Parsons et al. 2020; Sabetian et al. 2021). Through integrating microchemical otolith profiling with dynamic time warping (DTW)—an algorithm that measures similarity between temporal sequences—we aim to precisely identify the timing and nature of shifts in snapper behaviour and habitat utilisation over the past 800 years. This methodical investigation is designed to establish a solid foundation for enhanced ecosystem management and more sustainable conservation strategies, leading to the formulation of more accurate restoration goals.

## 2 Material and Methods

### 2.1 Model Species

The New Zealand snapper is a fish of significant ecological, economic, and cultural importance, belonging to the Sparidae family (Parsons et al. 2014). Juvenile snapper occupy sheltered, structured nearshore habitats, such as seagrass meadows, estuaries, and mussel beds, which provide essential shelter and food for early development (Parsons et al. 2014, 2016, 2020; Ross et al. 2007). This reliance on nearshore habitats during their first 6–12 months makes them particularly vulnerable to habitat degradation and other anthropogenic disturbances (Parsons et al. 2014). Once juveniles reach 6–7 cm in length, they begin migrating offshore to broader coastal areas, transitioning to habitats where structural dependence decreases (Parsons et al. 2013; Francis 1995).

Snapper are a demersal species that inhabit rocky reefs and sandy or muddy bottoms, typically at depths between 15 and 60 m, though they can be found as deep as 200 m (Parsons et al. 2011). Although generally solitary, they form spawning schools during spring and early summer (September–January/February) (Leach and Boocock 1994; Leach and Davidson 2000; Parsons et al. 2014), and are oviparous serial spawners, releasing multiple batches of eggs across a single season (Crossland 1977; Lodé 2012). In the Hauraki Gulf sexual maturity occurs between ages 2 and 5 years, with lengths ranging from 23 to 30 cm (Crossland 1981; Parsons et al. 2014). Snapper have a maximum recorded lifespan of 54 years and can grow to an average maximum length of 100 cm, though archaeological records suggest individuals may have historically reached up to 60 years (Kalish 1993; Leach 2006; Parsons et al. 2014).

Archaeological evidence identifies snapper as the most consumed fish species in the upper North Island prior to European settlement, as evidenced by Māori middens (Leach 2006). In modern times, snapper fisheries significantly contribute to New Zealand's economy, generating 160 million USD in direct gross output between 2010 and 2015 (Williams et al. 2017). Snapper are also a key recreational species, with the SNA1 fishery zone alone contributing a substantial portion of the total harvest. During the 2017–2018 fishing year they were the most landed species in the SNA1 zone, with recreational catches totalling 3467 t, over 62% of which came from the Hauraki Gulf (Hartill et al. 2019).

### 2.2 Study System

New Zealand's ecological diversity makes it an ideal place to explore the effects of human activities on ecosystems. Human activities, including swidden agriculture, logging, and urban development, have dramatically altered the country's ecological dynamics since its colonisation roughly 800 years ago. Early Polynesian settlers (Māori) had a profound impact on the environment (McWethy et al. 2014). They significantly reduced forest cover, with deforestation being complete in lowland Hawkes Bay, North Island, within 60–155 years after initial settlement in ca. 1280 CE (Holdaway et al. 2019). Modern‐day New Zealand scientists characterise this process as a significant transformation of the country's ecological landscape due to ancient human activities (McWethy et al. 2010).

Gibbs (2008) provides a good overview of New Zealand's fishing history: prior to about 1800, fishing activities were restricted to Māori subsistence fishing along many stretches of the New Zealand coastline. After a surge of European settlers, the period between 1790 and 1820 saw a sealing and whaling boom, and the fishing industry in New Zealand underwent significant changes, with the establishment of commercial steam trawling in 1882. In the following years, New Zealand experienced a significant increase in occupied productive land, expanding from approximately 8 million hectares in 1870 to 17 million hectares by 1911, reflecting the country's rapid land‐use change and development during this period (Álvarez et al. 2011). These historical shifts, marking New Zealand's localised Industrial Revolution, led to habitat fragmentation and loss, native animal extirpations, heavy fishing pressures, the introduction of invasive species, and increased chemical pollutants and turbidity in estuaries (Gibbs 2008; Matthaei and Piggott 2019; Wyse et al. 2018). Thus New Zealand emerges as an ideal field‐laboratory for assessing the effects of anthropogenic stressors on coastal fish populations and for formulating effective conservation strategies.

### 2.3 Otolith Assemblages: Archaeological, Historical, and Modern Snapper Samples

The SNA1 Fishery (SNA1), located along the east coast of New Zealand's North Island, encompasses the Hauraki Gulf, East Northland, and the Bay of Plenty. Managed as a single meta‐population by the New Zealand Ministry for Primary Industries (MPI), SNA1 reflects genetic connectivity and cohesive movement patterns, indicating that snapper in this region function as a unified stock (MPI 2016). SNA1 is one of New Zealand's most significant fisheries, contributing substantially to inshore catches. Management measures include Total Allowable Catch (TAC) limits, size restrictions, and seasonal controls, with the 2023 TAC set at 8050 t (commercial: 4500 t; recreational: 3050 t; customary: 50 t; other mortality: 450 t) (MPI 2023).

**FIGURE 1:**
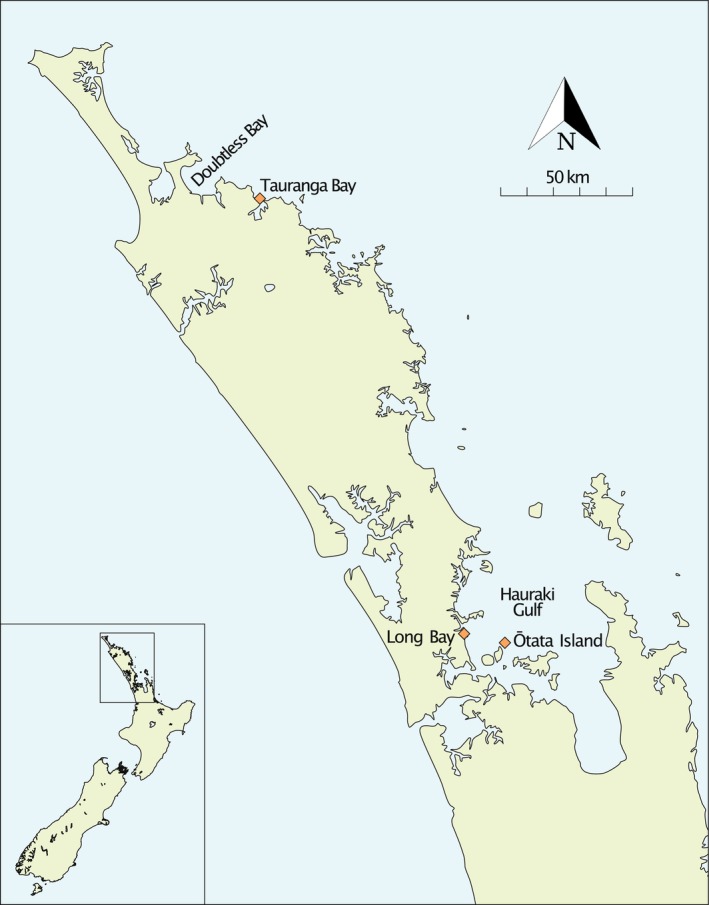
Sampling sites for otolith collection across Northland and the Hauraki Gulf, New Zealand.

To investigate long‐term ecological changes, we collected otolith samples spanning over eight centuries from three distinct sources. The archaeological samples (*n* = 63) were retrieved from pre‐European Māori middens, where fish remains were deposited following fishing and consumption. The historical samples (*n* = 20) were sourced from MPI's archival collection, originally gathered from commercial fisheries in the Hauraki Gulf in 1975. The modern samples (*n* = 130) were obtained from recreational fishers, with collections conducted in the Hauraki Gulf between 2016 and 2020, and in Doubtless Bay in 2020 (Figure 1 and Table 1).

**TABLE 1:** All otolith sampling sites are within the Snapper Auckland East (SNA1) management area as considered by the New Zealand Ministry of Primary Industries (MPI) for fishery management.

Sample location	Epoch	Origin of otoliths	Sampling size
Northland	13th–14th Century	Tauranga Bay, Archaeological midden	10
Hauraki Gulf	14th Century	Ōtata Island, Archaeological midden	5
Hauraki Gulf	15th Century	Long Bay, Archaeological midden	38
Hauraki Gulf	15th Century	Ōtata Island, Archaeological midden	5
Hauraki Gulf	17th–18th Century	Ōtata Island, Archaeological midden	5
Industrial Revolution			
Hauraki Gulf	20th Century	MPI collection	20
Hauraki Gulf	21st Century	Recreational fishing	60
Northland	21st Century	Doubtless Bay, Recreational fishing	70

### 2.4 Northland Samples

#### 2.4.1 Tauranga Bay (13th–14th Century CE)

Situated just outside Whangaroa Harbour's south head in Northland, the Tauranga Bay site (P04/639) yielded a rich midden containing shellfish, fish, bird, and terrestrial and sea‐mammal remains, along with various artefacts like bone fishhooks. Dating to the period of initial settlement in New Zealand in 13th to mid‐14th centuries, this site was primarily a fishing camp associated with more extensive settlement sites such as nearby Houhora. There was only a single occupation layer from which the otoliths were selected. The excavations have not yet been fully reported (Campbell 2004).

#### 2.4.2 Doubtless Bay (21st Century CE)

Doubtless Bay, situated on the east coast of New Zealand's North Island, served as a site for modern‐day otolith sample collection in Northland. These samples were obtained from recreational fishers.

### 2.5 Hauraki Gulf Samples

#### 2.5.1 Ōtata Island (14th, 15th, and 17th–18th Century CE)

The archaeological site on Ōtata Island in the Hauraki Gulf (R10/139) consists of four distinct cultural layers, each marked by shell middens containing fish and bird bones, as well as a variety of artefacts. We selected otoliths from three of the cultural layers at this site: Occupations 1, 3, and 4/5. The oldest, Occupation 1, dates to the 14th century, shortly after initial settlement and before the eruption of the nearby Rangitoto volcano around 1400 CE (Shane et al. 2013). This layer is overlain by a tephra deposit from Rangitoto and is followed by Occupation 3, which dates to the late 15th century. This occupational layer shows evidence of significant environmental changes, including deforestation, the emergence of shrubs and short‐lived tree species, and a decline in bird species. The youngest layer, Occupation 5, is dated to the 17th–18th centuries. The excavations at this site have not yet been fully reported (Furey and Ash 2020, 2021).

#### 2.5.2 Long Bay (15th Century CE)

The Long Bay Restaurant site (R10/13740) is located just north of Auckland city. Six stratified layers of shell midden, containing fish, bird, terrestrial, and marine mammal bones, along with a variety of artefacts—most notably shell fish hooks—were radiocarbon dated using Bayesian analysis, placing their occupation between 1430 and 1485. These occupations reflect changes in settlement patterns and resource procurement, particularly a transition from sourcing obsidian from distant locations, indicative of extensive social networks, to relying on more local sources (Campbell et al. 2019).

#### 2.5.3 Historical Samples (20th Century CE)

Historic otoliths were gathered as part of the MPI's earliest recorded sampling of commercial fisheries in the Hauraki Gulf in 1975. Although precise catch locations within the Gulf are not available, these samples provide a valuable historical perspective on snapper populations in the region.

#### 2.5.4 Inner Hauraki Gulf (21st Century CE)

Post‐processed snapper frames were collected from recreational fishers operating in the Hauraki Gulf on the eastern coast of New Zealand's North Island within the SNA1 snapper management area. Based on fishers' self‐reports, these samples were exclusively sourced from inner Hauraki Gulf locations, including the Whangaparāoa Peninsula and Rangitoto Channel. The samples were collected over the course of multiple fishing trips from various recreational fishers operating in the designated inner Gulf areas.

### 2.6 Otolith Element Analysis and Data Cleaning

Otoliths, calcified structures in a fish's inner ears, chronicle an individual's environmental history. As fish move between coastal waters and the open ocean, their otolith elemental compositions shift, reflecting the surrounding water chemistry (Campana and Thorrold 2001). Coastal waters, with their unique mix of nutrients and human influences, imprint distinct elemental signatures on otoliths compared to the more chemically consistent open ocean (Izzo, Reis‐Santos, and Gillanders 2018). Analysing these signatures provides insights into a fish's movement patterns and habitat preferences. Moreover, otolith data can highlight shifts in fish behaviour due to environmental stressors (Thomas and Swearer 2019) and offer a perspective on the health of fish stocks. Essentially, otoliths, with their daily accretion of calcium carbonate that isn't metabolically reworked after deposition, present a biological chronometer to analyse individual fish movement behaviour and habitat interactions (Palumbi et al. 2003; Thomas et al. 2017).

All otoliths were washed, cleaned in 40% ethanol, and air‐dried overnight. Archaeological otoliths underwent an additional cleaning step, where they were placed in a vial with alcohol and shaken at 3000 rpm in a Vortex Mixer for 1 min to dislodge any dirt or organic matter. Following cleaning, all otoliths were weighed using a Mettler AC 100 fine scale with a precision of ±0.001 g. Otoliths were then ground on both sides to create a thin transverse section of the centrum, which was used for both age determination and chemical analysis.

Age determination was conducted on the thin transverse sections of sagittal otoliths. The otoliths were ground on both sides until the cross section with the core (the ‘middle’ of the otolith) was exposed. Each otolith was mounted on the edge of a glass slide using Crystalbond 509 glue and ground down to the edge of the slide with a 3000‐grit diamond‐encrusted disc. The otoliths were then placed face down on another slide, and further ground until a thin transverse section was achieved. Opaque growth rings, appearing as dark bands under transmitted light, were counted to estimate annual age. The formation of these rings, due to slower growth during the winter, has been validated in snapper otoliths (Francis, Paul, and Mulligan 1992).

The age readings are considered conservative, as they were based on visible increments, potentially underestimating the true age of the fish. This was particularly challenging for older specimens, whose otoliths exhibited highly compacted annual increments at their margins, complicating accurate age determination. For statistical analysis, an Analysis of Covariance (ANCOVA) was conducted to examine the relationship between otolith mass (g) and age (years) across different assemblages. After diagnostic checks of the ANCOVA model, including assessments of residual normality, homoscedasticity, and linearity, no substantial violations of model assumptions were identified, and thus no data transformations were applied to preserve the interpretability of the results. Post hoc pairwise comparisons of slopes were performed using the *emmeans* package in R to evaluate differences in regression slopes among assemblages.

We employed laser ablation inductively coupled plasma mass spectrometry (LA‐ICP‐MS) to measure elemental compositions along an oblique ablation path (‘dog leg’) from the core to the proximal tip of each otolith. Before ablation, the surface of each otolith was polished with 5‐μm Al lapping paper in distilled water. Sectioned otoliths were mounted on microscope slides and analysed using a quadrupole ICP‐MS connected to a 193 nm Resonetics (now Applied Spectra) RESOlution LR laser ablation system, operated in a helium atmosphere with a flow rate of 300 mL/min, mixed with 3.5 mL/min high purity N₂ and ~1.0 L/min Ar. To ensure stability, the argon background was purged 24 h prior to measurement.

For pre‐ablation, we used a 75 μm spot size at 40 μm/s, followed by a main ablation with a 50 μm spot size at 10 μm/s; both ablations operated at a fluence of 2.5 J/cm^2^ with a firing frequency of 10 Hz. A pre‐ablation cleaning pass was conducted to remove surface contaminants, followed by a 25‐s pause to measure the background before the main ablation. Calibration was performed against NIST610 glass standard, with NIST612 and USGS MACS3 used as controls. The Agilent 7900 quadrupole ICP‐MS was optimised to minimise oxide (156/140) and double charge (22/44) formation rates (< 0.25%) while ablating NIST612 standards, with adjustments to the argon flow to keep the Th/U signal ratio below 115%.

The isotopes collected were Li^7^, Na^23^, Mg^24^, Al^27^, P^31^, K^39^, Ca^43^, Mn^55^, Cu^63^, Zn^66^, Rb^85^, Sr^88^, Ba^138^ and Pb^208^. Data reduction was conducted using Iolite v3.71, applying the ‘X_Trace_Elements_IS’ scheme using a default Ca content of 40.08% w/w (assuming CaCO₃). This scheme extracts Ca concentrations from the reference files for NIST610, NIST612, and USGS MACS3. Results were exported as time‐resolved concentrations at the cycle level in μg/g and subsequently converted to mmol/mol Ca. Precision was consistently within 2% RSD for NIST612 and under 6% RSD for USGS MACS3.

Otolith transects were cleaned of aberrant isotopic signatures on the otolith edges by filtering the intensity of the ion beam in counts‐per‐second (cps) for the most abundant isotope, Ca^43^. All isotopic measurements below 15,000 cps were removed, a value which corresponded to the lower tail of the values measured throughout the study (mean counts per second [CPS] of Ca^43^ was 29,766 CPS throughout the study). This removed any drops or rises in the element/Ca values due simply to low sample detections at the edge of the otolith or surface defects (cracks glues etc.) which could change the shape of the data. All raw ablation data can be found in Lilkendey et al. (2024).

To address potential diagenetic alterations from burial or excavation handling, which could substitute biological elements with contaminants, we implemented strict data‐cleaning protocols. We trimmed the first 5 s and the last 10 s of all otolith ablation data to remove any potential contamination. Elemental transects were standardised to a maximum length of 3000 μm, encompassing the juvenile and adult periods for most specimens (mean transect length: 2935 μm). This approach ensured that our chemical profiles were representative of environmental conditions during the fish's lifespan while minimising bias from post‐depositional or life‐history effects. These precautions strengthen the reliability of our interpretations regarding habitat shifts and ecological changes across time periods.

In archaeological contexts, traditional Māori cooking methods, such as the hāngī (earth ovens, often with heat retainer rocks), were commonly employed to prepare fish, which exposed otoliths to heat prior to burial (Whyte et al. 2001). However, studies such as Avigliano et al. (2020) have demonstrated that exposure to heat does not significantly affect Sr or Ba concentrations, thereby reinforcing the reliability of these elements as environmental proxies even in otoliths subjected to cooking processes. To address concerns about diagenetic alterations, such as those highlighted by Miller et al. (2011), we implemented several measures to ensure the validity of the data. Burnt otoliths were identified based on discoloration or surface cracking, and only two such samples were detected; these were excluded from the study. Additionally, all archaeological otoliths underwent an extra cleaning step involving agitation in 40% ethanol at 3000 rpm in a Vortex Mixer to remove potential contaminants introduced during burial or excavation.

To mitigate diagenetic effects that might not be visually apparent, such as elemental substitution by environmental contaminants during burial (Cook et al. 2015), we trimmed the first 5 s and the last 10 s of all otolith ablation data. Elemental transects were also standardised to a maximum length of 3000 μm to encompass the juvenile and adult periods for most specimens (mean transect length: 2935 μm). This precaution prevented bias from longer‐lived fish, which were primarily from archaeological contexts dating to the 14th–18th centuries. Including their full life history could otherwise cause clustering effects that obscure temporal trends in movement or salinity conditions. Collectively, these measures ensured that the chemical profiles analysed were representative of original environmental conditions, enabling a valid comparison between archaeological and modern otoliths.

Following trimming, all transects were smoothed using three‐pass outlier detection using the *tsclean* function in the *forecast* package for R (Hyndman and Khandakar 2008) followed by a 100‐point moving mean. Transects were then re‐interpolated to the mean length of all transects (1392 points).

### 2.7 Analysis of Continuous Otolith Microchemical Data via Dynamic Time Warping

The research field of otolith chemistry has been hampered by the conventional point analysis multi‐tracer methodological approach, which focuses on the geochemistry of specific time points in a fish's life. This approach cannot unlock the richness of continuous time‐series data nor the details of temporal autocorrelated structure in the values of elements. Furthermore, studies are investigating the application of sophisticated time‐series techniques to infer fish movement and habitat quality over time (Sabetian et al. 2021). One such approach to identify similarities in movement patterns between individuals from continuous data, such as otolith microchemical profiles, is DTW (Arai et al. 2024).

Originating as a method for speech recognition where variations in speed and accent created phase shifts in spoken words (Myers and Rabiner 1981), DTW has since evolved into a popular tool for time series comparison, owing to its ability to effectively match time series data that may have temporal misalignments (Rakthanmanon et al. 2012). Further advancements have enabled its application in conjunction with existing clustering and classification methods (Sardá‐Espinosa 2017). Leveraging otolith microchemistry alongside DTW provides an innovative means to assess fish temporal habitat use, even when other methods struggle with temporal inconsistencies (Hegg and Kennedy 2021).

We clustered our ICP‐MS data based on the shape of the multivariate laser transect data using DTW. Data was first cleaned and smoothed, then trimmed to the juvenile and early adult period. Because DTW acts only on the shape of the data, with isotopic magnitude removed by z‐scaling, otoliths were subjected to exploratory clustering on whole transect multivariate means to ensure that no important information was lost in the magnitude of each isotopic transect. Individual life histories for all fish were then clustered hierarchically based on the multivariate DTW distance to identify unique life history groups.

Since DTW requires z‐normalisation of the time‐series, it can obscure meaningful differences in life‐history transects whose shapes are similar but whose element/Ca magnitudes differ. To evaluate whether elemental scale differences influenced potential groupings, exploratory clustering was first conducted on multivariate whole‐transect means to identify patterns based on element/Ca magnitude. This clustering on whole otolith means was done using the *Mclust* package for model‐based clustering (Scrucca et al. 2016) and applied to the mean of the full multivariate elemental data across the entire transects. The clustering algorithm selected a three‐cluster solution based on maximising Bayesian Information Criterion (BIC). However, the clusters contained large overlaps and no specific effects of isotopic magnitude that justified using them to separate life histories. Therefore, DTW analysis proceeded on the entire dataset without any pre‐clustering based on transect means.

DTW distance was calculated on the smoothed and re‐interpolated transects after z‐scaling, without the Keogh lower bound, and with a window size of 100. Results were hierarchically clustered using Wards distance. Cluster solutions were evaluated using silhouette distance.

The element/Ca ratio across the 3000 μm standardised length of each otolith was compared between DTW cluster solutions for common metals; Al/Ca, Cu/Ca, Li/Ca, Pb/Ca, Zn/Ca. Clusters were compared using Wilcoxon ranked‐sum tests with Bonferroni correction to maintain a family‐wise error rate of *α* = 0.05.

## 3 Results

### 3.1 Otolith Growth

The pairwise comparisons of regression slopes reveal significant differences in otolith growth patterns among assemblages, demonstrating temporal variability in the relationship between otolith mass and age (Appendix S1: Figure 1). Otolith growth rates in the Northland Tauranga Bay samples from the 13th to 14th century were significantly lower compared to all other assemblages, including those from the Hauraki Gulf across historical and modern periods, as well as the modern Northland Doubtless Bay samples. Specifically, the slopes in Tauranga Bay were shallower than those observed in the 14th‐century Ōtata Island midden, the 15th‐century middens from Ōtata Island and Long Bay, and the modern samples from the 20th and 21st centuries. However, the interpretation of findings from Tauranga Bay should be approached cautiously, as the archaeological otoliths from this site were particularly challenging to read, with compact growth increments and high variability potentially influencing the observed trends.

Within the Hauraki Gulf, slopes from the 14th and 15th centuries, including samples from Ōtata Island and Long Bay, were similar, with no significant differences observed between these periods. Modern samples, including those from the 20th and 21st centuries, displayed steeper slopes compared to earlier assemblages, indicating increased otolith growth rates over time (Appendix S1: Table 1).

### 3.2 Hierarchical Clustering

**FIGURE 2:**
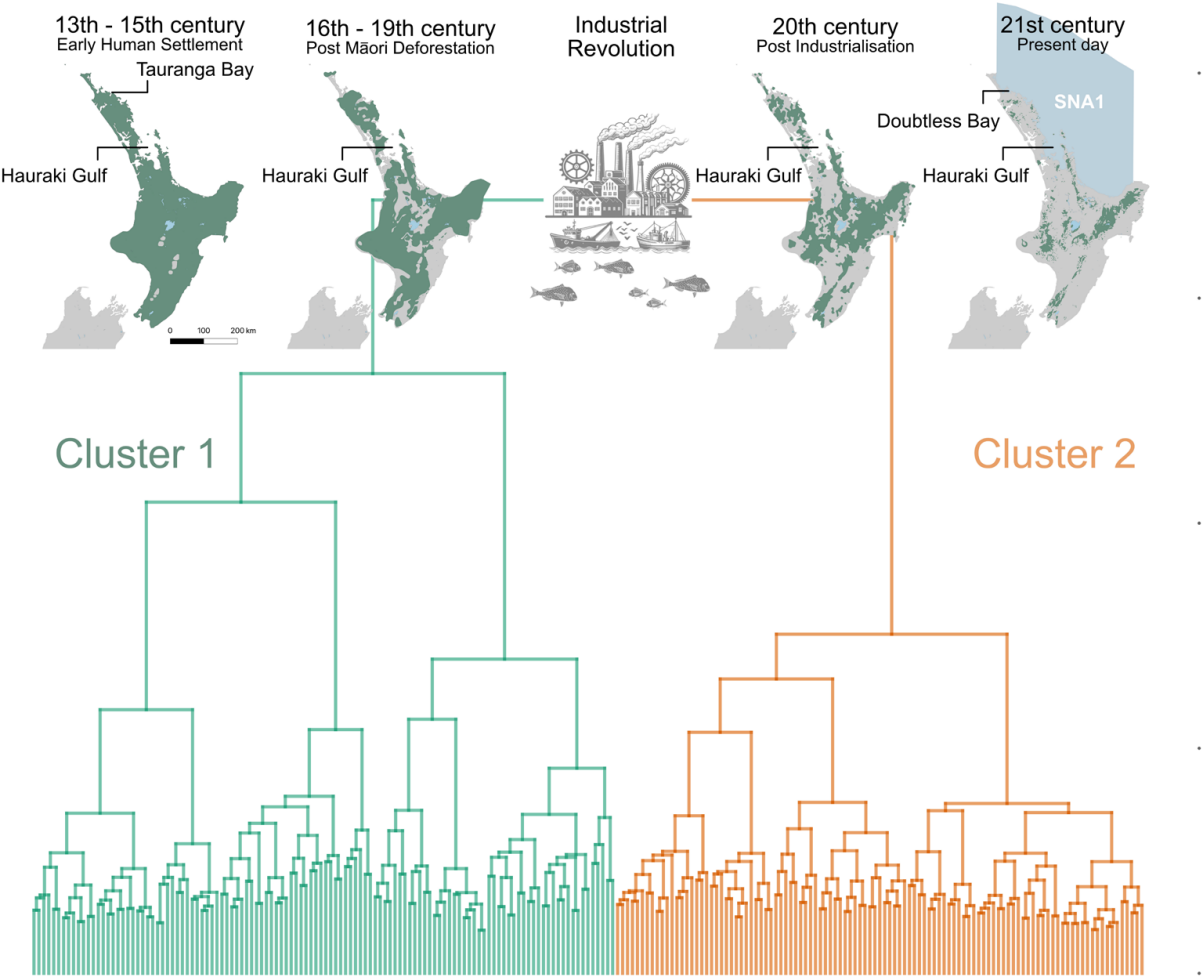
Timeline of human impacts on New Zealand’s forest and coastal ecosystems, spanning early human settlement (13th–15th century), post‐Māori deforestation (16th–19th century), the Industrial Revolution (19th–20th century), post‐industrialisation (20th century), and the present day (21st century). Dendrograms illustrate hierarchical clustering of New Zealand snapper *Chrysophrys auratus* otolith chemical profiles via dynamic time warping (DTW), with branch heights indicating DTW distances and chemical similarity across samples. Maps highlight sampling locations within the Snapper Auckland East (SNA1) stock management area, including the Hauraki Gulf, and Northland (Tauranga Bay and Doubtless Bay). Native forest cover (green) changes on the North Island are redrawn from Weeks, Overton, and Walker (2012) and Wyse et al. (2018).

The hierarchical clustering of otolith chemical profiles via DTW revealed distinct patterns in snapper behaviour across different time periods. The silhouette index—a measure used to interpret and validate the consistency within clusters of data—ranged from 0.018 to 0.062, indicating some level of differentiation but also significant overlap among groups. Cluster solutions with 2, 3, and 4 clusters had the highest silhouette indices (0.053, 0.062, 0.051 respectively), with cluster numbers of 5 through 8 all lower, ranging from 0.013 to 0.025. This modest separation was further corroborated by the vertical distances between branches on the hierarchical tree, suggesting that a bifurcated grouping was both conservative and rational based on the dendrogram hierarchy (Figure 2).

### 3.3 Otolith Microchemical Profiles

**FIGURE 3:**
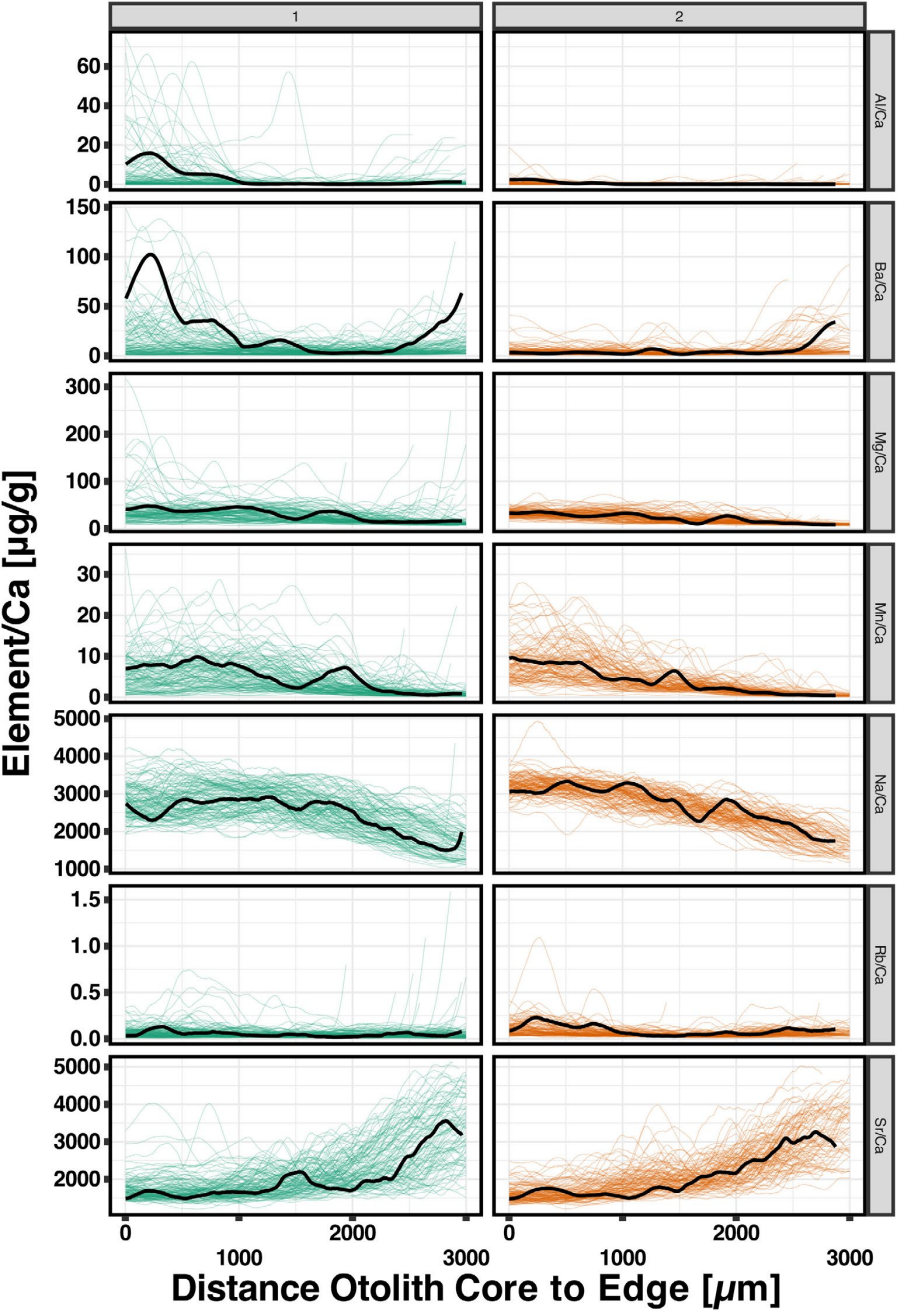
Element‐to‐calcium ratios (element/Ca) for various elements measured along transects from the otolith core to the edge for two clusters of New Zealand snapper *Chrysophrys auratus* otoliths. Each subplot represents a different element (aluminum [Al], barium [Ba], magnesium [Mg], manganese [Mn], sodium [Na], rubidium [Rb], and strontium [Sr]) and shows how their concentrations change with distance from the otolith core. The data are divided into two clusters, with Cluster 1 and Cluster 2 represented by different colors. The centroid plots (black) illustrate the most representative  elemental concentration profile for each cluster based on dynamic time warping distance.

Notably, the centroid plots reveal that otolith element‐to‐calcium ratios (element/Ca) in Cluster 1 exhibit higher Al and Ba values early in life, whereas Cluster 2 displays a Ba peak later in life. Cluster 1 otoliths show more variable Mg and Mn profiles, with Mn fluctuations occurring later in life compared to Cluster 2. Additionally, Cluster 1 otoliths demonstrate a delayed onset of the downward trend in Na, with greater variability in values among individuals, and a slightly later onset of increasing Sr. These distinct temporal patterns in element/Ca ratios between Cluster 1 and Cluster 2 across the otolith transects underscore significant differences in life‐history dynamics (Figure 3).

**FIGURE 4:**
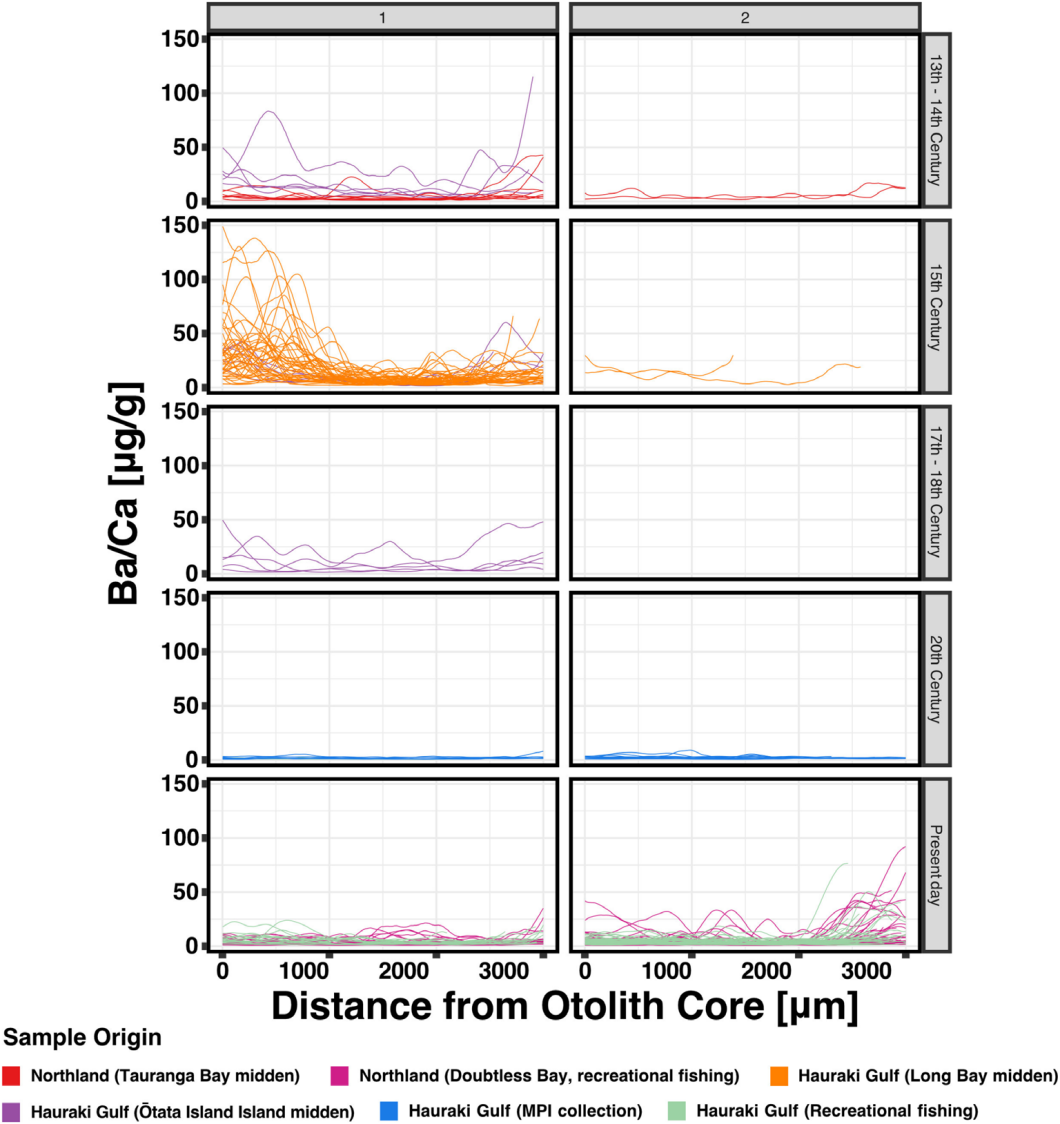
Barium‐to‐calcium (Ba/Ca) ratios in the otoliths of New Zealand snapper ( *Chrysophrys auratus* ) obtained from archaeological Māori middens, historical fisheries surveys, and contemporary recreational fisheries. The *y*‐axis represents Ba/Ca ratios, while the *x*‐axis depicts the transect length across the otolith, corresponding to the fish's lifetime from core to edge. The plot shows the clustering of Ba/Ca profiles based on dynamic time warping distances (columns) and separates the data by source and epoch (rows), providing a temporal perspective on Ba/Ca variation in these fish. MPI, New Zealand Ministry of Primary Industries.

Our analysis of otolith chemical profiles further demonstrated a clear shift in life history across these temporal and spatial contexts. In the 13th–14th and 15th centuries, all but four individuals from the Hauraki Gulf clustered in Cluster 1, characterised by high Ba/Ca ratios early in life. These included two individuals from the 14th‐century Ōtata Island midden and two from the 15th‐century Long Bay midden. All individuals from the 17th–18th‐century Ōtata Island midden also aligned with Cluster 1. By contrast, the majority of fish from the 20th and 21st centuries, including modern samples from the Hauraki Gulf and Doubtless Bay, clustered in Cluster 2. This group was distinguished by the absence of a Ba/Ca peak early in life and a smaller late‐life Ba/Ca peak (Figure 4).

### 3.4 Cluster Distribution

**FIGURE 5:**
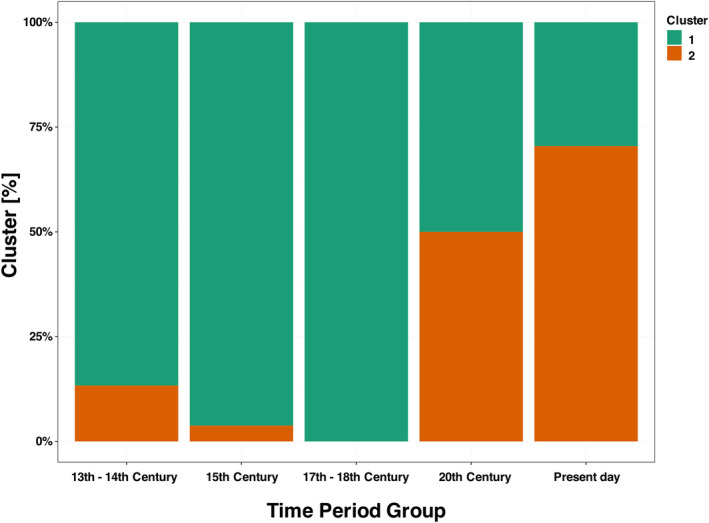
The temporal succession of cluster distributions of fish behavioural patterns in New Zealand snapper *Chrysophrys auratus* exhibits a tipping point in the 19th century. The ordinate axis shows the percentage of otolith microchemical profiles allocated to each of the two clusters.

During the 13th and 14th centuries, the cluster distribution of all elemental profiles combined was characterised by 80% to Cluster 1 and 20% of the samples grouped in Cluster 2. This distribution remained stable in the 15th century, with 95% of samples associated with Cluster 2 and only 5% with Cluster 2. In the 17th and 18th centuries, while the available data from five samples showed a continuation of the trend with all samples falling into Cluster 1, the limited sample size of 5 otolith profiles cautions against overstatement of this finding. The 20th century marked an increased representation of Cluster 2, with an equitable distribution of 50% of samples in both clusters. In the 21st century samples, Cluster 2 defines the majority with 70% of the samples, while Cluster 1 encompasses 30% (Figure 5).

### 3.5 Comparison of Trace Metals Between Clusters

**FIGURE 6:**
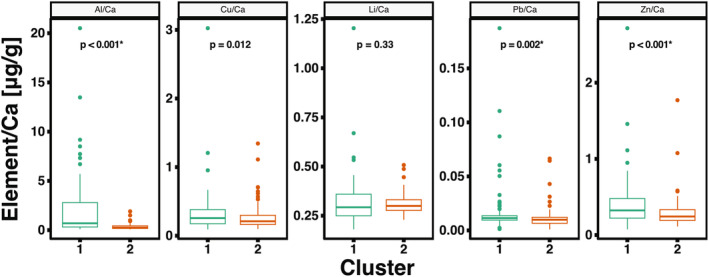
Boxplots showing element‐to‐calcium ratios (element/Ca) for aluminum (Al), copper (Cu), lithium (Li), lead (Pb), and zinc (Zn) in snapper *Chrysophrys auratus* otoliths. Comparisons are made between the 13th–19th centuries (Cluster 1) and the 20th century to the present (Cluster 2). Statistically significant differences (family‐wise *α* = 0.05, Bonferroni corrected *p* < 0.01, indicated with*) indicate temporal shifts in elemental exposure.

Element‐to‐calcium ratios (element/Ca) in snapper otoliths revealed significant temporal variation across epochs: the 13th–19th centuries (Cluster 1) and the 20th century to the present (Cluster 2). Elevated levels of Al, Cu, Pb, and Zn in Cluster 1 otoliths suggest greater historical reliance on freshwater or estuarine environments compared to Cluster 2 individuals (Figure 6).

## 4 Discussion

Coastal ecosystems worldwide are undergoing significant transformations due to anthropogenic pressures, yet SBS often masks the full extent of these changes, obscuring historical benchmarks and impeding effective management. Our study reveals a profound shift in habitat‐use behaviour by juvenile snapper in New Zealand's coastal waters over the past eight centuries. Historically, juvenile snapper thrived in low‐salinity estuarine nurseries, but modern populations predominantly inhabit higher‐salinity marine environments. This transition aligns with increasing sedimentation and turbidity driven by land‐use changes, with New Zealand's localised Industrial Revolution serving as a tipping point. Observed across an entire stock unit, this systematic alteration of estuarine ecosystems underscores the reorganisation of snapper populations, driven by environmental degradation and anthropogenic pressures, reflecting widespread ecological transformations with global parallels.

### 4.1 Otolith Growth and Temporal Comparisons

Disentangling the physiological influences from environmental effects on otolith composition is a challenging task, compounded by the dynamic nature of aquatic ecosystems (Reis‐Santos et al. 2023). Otolith growth provides a valuable lens for examining environmental and ecological influences on fish populations, as these structures grow incrementally in tandem with somatic development (Campana and Thorrold 2001). In modern snapper, growth rates have declined, particularly in the recovering Hauraki Gulf, where density‐dependent competition, temperature variability, and fluctuating food availability impose constraints (Parsons et al. 2014). Elevated somatic growth would result in compressed otolith growth increments, complicating direct comparisons between historical and modern samples by potentially amplifying or obscuring temporal differences in otolith chemistry. Notably, our samples reveal spatial and temporal variability in otolith growth patterns, with slower growth trends in pre‐industrial periods contrasting with the more rapid growth observed in modern assemblages. These altered growth dynamics underscore the influence of changing environmental conditions over time ‐ emphasising the importance of considering the timeframe in interpretation.

To address these challenges, we employed DTW to align otolith elemental profiles based on temporal patterns rather than spatial positioning. This approach ensures robust comparisons despite variability in otolith size or growth rates (Hagen and Harper 2023; Hegg and Kennedy 2021). DTW measures the amount of warping needed to align each time series, distinguishing the unique shapes created in otolith chemical transects by the pattern of physical location and life‐stage timing (Secor et al. 2020). While metabolic changes can influence absolute elemental concentrations (Campana 1999), DTW enables us to focus on the relative timing of shifts in elemental ratios, which are less sensitive to growth‐related and physiological effects. By preserving the temporal integrity of chemical profiles, we can identify genuine ecological shifts, such as changes in habitat use, while minimising the influence of metabolic or ontogenetic factors.

While some complexities may arise from shifts in ontogenetic milestones and the variability within archaeological samples, which may encompass individuals collected across decades or even centuries, DTW mitigates these challenges by focusing on the alignment of temporal patterns. This enables meaningful comparisons of otolith chemistry across epochs and regions, including Northland and the Hauraki Gulf. Our approach ensures that observed differences in elemental concentrations are reflective of genuine ecological shifts rather than artifacts of ontogenetic or spatial variability. The application of DTW reinforces the robustness of our findings across the SNA1 stock, providing a powerful framework for disentangling temporal and growth‐related effects while elucidating long‐term ecological changes.

### 4.2 Environmental Degradation and Juvenile Snapper Habitat Shifts

Human activity has left an indelible mark on ecosystems worldwide, with coastal environments experiencing some of the most severe impacts (Lu et al. 2018). In New Zealand estuaries, deforestation, agricultural intensification, and urbanisation have caused sedimentation rates to soar (Thrush et al. 2008). These changes have pushed juvenile snapper away from historically critical estuarine habitats into high‐salinity marine environments (Campbell et al. 2021; Sabetian et al. 2021). Elevated turbidity, which impairs larval foraging and settlement success, emerges as a key mechanism behind this habitat shift (Lowe, Morrison, and Taylor 2015). Such transformations mirror trends observed in terrestrial ecosystems, where land‐use changes and climate warming have disrupted biodiversity and community structure (Montràs‐Janer et al. 2024).

The chemistry of fish otoliths provides valuable insights into past and present environmental conditions, particularly for tracing habitat use during early life stages. While otolith chemistry alone cannot precisely pinpoint the geographical location of juvenile snapper nurseries, consistent changes in elemental signatures reveal critical shifts in habitat use over time. Ba/Ca ratios, a well‐established indicator of low‐salinity environments, are particularly informative in estuarine ecosystems, where Ba concentrations are elevated due to freshwater influx and limited exchange with marine waters (Elsdon and Gillanders 2005a). Historically, estuarine nurseries played a vital role for juvenile snapper, as evidenced by high Ba/Ca ratios in their otoliths, a pattern that aligns with findings in other estuary‐dependent species (Gillanders and Kingsford 2002).

Our findings reveal a pronounced decline in Ba/Ca ratios in modern otoliths, reflecting a widespread reduction in the use of low‐salinity estuarine habitats. This decline is likely driven by anthropogenic impacts such as sedimentation and habitat loss, which have degraded estuarine ecosystems and pushed juvenile snapper to rely more heavily on high‐salinity marine habitats (Morrison et al. 2009). Hamer, Jenkins, and Coutin (2006) demonstrated that Ba/Ca ratios in otoliths of snapper strongly reflect environmental Ba levels, with significantly higher ratios in estuarine habitats compared to marine environments, highlighting the significant influence of estuarine Ba enrichment on otolith chemistry. In contrast, modern otoliths in our study show consistently low Ba/Ca ratios, even though North Island New Zealand's estuaries and bays should exhibit much higher Ba concentrations due to natural and anthropogenic enrichment (Huteau 2017). The absence of high Ba/Ca ratios in modern otoliths underscores the near‐total avoidance of estuarine habitats by contemporary snapper populations. The contrast between historical and modern otolith chemistry further highlights these shifts: historical otoliths exhibit higher Ba/Ca ratios in early life, indicative of prolonged residency in estuarine nurseries, followed by a shift to marine habitats later in life. In contrast, modern otoliths display reduced Ba/Ca ratios throughout, suggesting a greater reliance on high‐salinity marine environments during all life stages.

While both Ba/Ca and Sr/Ca are influenced by salinity, their incorporation into otoliths reflects different environmental dynamics. Ba is more sensitive to short‐term salinity fluctuations, making it a robust proxy for low‐salinity estuarine environments (Elsdon and Gillanders 2005b; Gillanders 2005). In contrast, Sr tends to peak when fish transition from freshwater or estuarine environments to marine habitats, where salinity and Sr concentrations are naturally higher (Elsdon and Gillanders 2005b; Gillanders and Kingsford 2002). Sr/Ca ratios, however, tend to plateau or fluctuate more gradually, influenced by a broader range of factors such as temperature and duration of exposure, which can obscure their salinity signal (Elsdon and Gillanders 2005a). Together, Ba/Ca and Sr/Ca signals reveal critical shifts in juvenile habitat use, distinguishing historical reliance on estuarine nurseries from modern preference for high‐salinity marine environments. These findings underscore the importance of integrating multiple trace element proxies to better understand the ecological consequences of habitat degradation and shifts in habitat use.

### 4.3 Supporting Evidence From Trace Metal Analysis

The Ba‐based evidence is corroborated by patterns observed in other elemental markers, reflecting the environments in which fish lived throughout their lifespan (Thomas et al. 2017). Specifically, Ranaldi and Gagnon (2010) demonstrated the environmental sensitivity of trace metal incorporation in snapper otoliths, underscoring their value as environmental archives for historical and modern habitat conditions. For example, Al is known to decline with increased salinity, and Al/Ca shows decreasing trends in our modern samples, consistent with reduced use of low‐salinity, high‐nutrient estuarine waters (Mackin and Aller 1984; Xu et al. 2002).

Conversely, Mn/Ca increases in modern samples, aligning with higher salinity habitat use, consistent with findings that Mn concentrations in coastal waters increase with salinity (Mohan and Walther 2015). Cu/Ca and Pb/Ca profiles in archaeological samples align with patterns typical of stratified estuaries, offering further evidence of historical estuarine habitat use. Cu, for instance, exhibits a bell‐shaped curve as its binding behaviour changes with salinity (Louis et al. 2009; Ruan et al. 2024). Similarly, Pb concentrations peak at the freshwater–seawater interface in stratified estuaries before declining with dilution in seawater (Marcinek et al. 2022; Thouvenin et al. 2007). These findings are supported by Arslan and Secor (2005), who analysed Mn/Ca, Cu/Ca, and Pb/Ca ratios in otoliths of American eels from the Hudson River estuary, demonstrating significant differences across salinity zones and varied environmental exposures, further validating the utility of these markers in palaeo‐ecological studies.

Taken together, these multi‐element profiles provide compelling evidence that historical snapper populations relied more extensively on low‐salinity estuarine environments. In contrast, modern snapper exhibit a marked shift toward higher‐salinity marine habitats, reflecting substantial changes in habitat use over time.

### 4.4 Spatial Variability and Stock Cohesion

Understanding the spatial and genetic variability of fish populations is crucial to interpreting ecological shifts, as location‐specific differences in water chemistry and movement behaviours can significantly influence otolith signatures (Elsdon et al. 2008; Gillanders 2005). Our study leverages the cohesive nature of the SNA1 stock, which spans northern New Zealand, including the Hauraki Gulf, Northland, and the Bay of Plenty, as a genetically connected meta‐population (MPI 2016; Parsons et al. 2014). This genetic connectivity suggests that snapper across these regions share similar ecological characteristics, reducing the likelihood that observed chemical differences are driven solely by localised environmental factors. Modern samples were collected from locations overlapping with traditional Māori fishing grounds, ensuring spatial and temporal comparability between historical and contemporary datasets (Leach 2006; Whyte et al. 2001). This overlap ensures that both historical and modern samples reflect similar ecological contexts, minimising potential biases from location‐specific differences.

Snapper exhibit diverse movement behaviours, with some individuals showing high site fidelity as residents in reef or estuarine habitats, while others adopt migratory patterns, traversing a range of inshore and offshore environments depending on habitat availability and environmental conditions (Parsons et al. 2011). Acknowledging this behavioural diversity, our findings reveal that most individuals—whether migratory or resident—show diminished low‐salinity signatures in modern otoliths, suggesting that this pattern is widespread and not restricted to a specific geographic area. This population‐wide reduction underscores the systemic alterations to estuarine ecosystems that have fundamentally reshaped habitat dynamics across the SNA1 stock. The inclusion of modern samples from Northland (Doubtless Bay) and the Hauraki Gulf, both predominantly clustering in Cluster 2, highlights the widespread nature of these ecological changes. By analysing otolith chemistry across broad temporal and spatial scales, we demonstrate that shifts in juvenile habitat use reflect systemic degradation in estuarine ecosystems, rather than localised spatial variability.

### 4.5 Challenges of Habitat Adaptation and Recruitment Failure

The mismatch between historical and contemporary juvenile habitat use raises critical questions about snapper life history and adaptation. Interestingly, some Cluster 2 otoliths exhibit Ba/Ca peaks later in life, a pattern that likely reflects adult snapper returning to low‐salinity environments, possibly for spawning. This presents a potential paradox: if adults are spawning in low‐salinity habitats, why are juveniles not utilising these areas as they historically did? Several hypotheses could explain this phenomenon. Estuarine seagrass beds, critical nursery habitats for juvenile snapper, have suffered significant degradation due to siltation and other anthropogenic stressors (Morrison et al. 2009). The loss or degradation of these habitats may compel juveniles to seek alternative, higher‐salinity environments.

Another key hypothesis is recruitment failure in turbid estuaries. While direct evidence of recruitment failure remains limited, degraded estuarine habitats and increased turbidity strongly suggest reduced juvenile survival and settlement success. Turbidity reduces visibility, impairing larval foraging success and increasing mortality during critical early life stages (Lowe, Morrison, and Taylor 2015; Wenger et al. 2013). Murphy (2012) demonstrated that prey availability and foraging success play a key role in larval growth and survival, with slower growth increasing susceptibility to predation and starvation. Additionally, in northern New Zealand, environmental variables such as temperature, tidal ranges, and onshore winds influence larval settlement success, reflecting the sensitivity of snapper recruitment to changing estuarine dynamics (Sim‐Smith, Jeffs, and Radford 2013). This recruitment bottleneck could explain the absence of juvenile snapper in low‐salinity estuaries despite evidence of adult spawning. Juveniles that do survive under such conditions may experience reduced growth rates and higher mortality risks, further diminishing their fitness and representation in modern populations (Brown et al. 2017; Wenger, Johansen, and Jones 2012). These intertwined factors likely act in concert, producing the observed shift in juvenile snapper habitat use. The interplay between adult spawning behaviour and juvenile recruitment underscores the need for integrated management approaches that account for environmental degradation, larval survival, and habitat restoration to sustain snapper populations in changing coastal environments.

### 4.6 Overcoming Shifting Baselines

The phenomenon of shifting baselines further complicates our grasp of these changes, illustrating how successive generations adjust their perceptions of what constitutes a ‘healthy’ environment, thereby masking the true scale of human impacts on once untouched natural landscapes (Soga and Gaston 2018). The concept of shifting baselines, as outlined by Pauly (1995), illustrates how generational perceptions can normalise depleted stock sizes, obscuring the urgency of conservation efforts and emphasising the need for comprehensive historical data. Despite anecdotal evidence pointing to a significant decline in New Zealand snapper populations over the past century, the absence of comprehensive historical data has clouded perceptions of past ecosystem health (Parsons et al. 2009). This has resulted in a shifted baseline, where the absence of juvenile snapper in key nursery habitats appears paradoxical, given these habitats are perceived as healthy. This phenomenon has led to misconceptions about the sustainability of the SNA1 snapper stock sizes, masking potential declines and complicating conservation efforts (Parsons et al. 2020). Our findings underscore a fundamental shift away from low‐salinity estuarine nurseries toward high‐salinity marine environments, reflecting the degradation of these critical habitats. This shift challenges prevailing assumptions about the sustainability of current stock sizes, highlighting the urgent need to integrate historical baselines into conservation strategies.

### 4.7 Broad Implications for Coastal Ecosystem Management

Our study utilises continuous otolith microchemical profiles to track a shift in snapper movement patterns from clusters representing historically stable ecological conditions to those influenced by altered environments across 250 km, spanning Northland Bays and the Hauraki Gulf. The analysis reveals that the observed changes in snapper behaviour are more closely tied to temporal shifts than to specific geographic locations. By employing DTW, we identified consistent temporal patterns, indicating that the reorganisation of habitat‐use behaviour has occurred systematically across the SNA1 stock.

The clustering of life‐history patterns before the 19th century and another dominant pattern after the 19th century reflects the influence of widespread anthropogenic impacts, particularly sedimentation and habitat degradation. These shifts would be challenging to discern using traditional methods that divorce chemical data from temporal context, underscoring the utility of DTW in assessing long‐term ecological changes.

By integrating contemporary findings with palaeo‐ecological baselines and monitoring habitat health indicators derived from otolith analyses, we provide actionable insights for ecosystem management (Leonhard and Agiadi 2023). Indicators like the behavioural patterns of coastal fishes can help assess the impacts of stressors such as pollution, climate change, and habitat destruction on marine biodiversity. Our methodology transcends the limitations of SBS, offering a concrete measure of ecological recovery (Guerrero‐Gatica, Aliste, and Simonetti 2019). The historical perspective provided by this research serves as a foundation for policymakers to establish scientifically informed baselines, enabling the development of sustainable management strategies for snapper populations and coastal ecosystems worldwide.

## 5 Conclusions

Anthropogenic pressures have profoundly reshaped coastal ecosystems worldwide, with implications for biodiversity, fisheries, and ecosystem services. Our findings reveal how sedimentation and habitat degradation have fundamentally altered snapper nursery habitats over centuries, underscoring the long‐term ecological consequences of human activity. By employing trace element analysis and DTW methodologies, this study provides a replicable framework for assessing ecological shifts in coastal ecosystems globally. Importantly, our results highlight the phenomenon of SBS, wherein generational shifts in perception mask the true scale of historical degradation, complicating conservation efforts. Integrating palaeo‐ecological datasets with contemporary monitoring is essential for addressing SBS, enabling policymakers to establish realistic baselines for ecosystem health and fisheries management. Our research supports the implementation of stricter regulations on land use and agricultural practices to mitigate sedimentation and estuarine habitat loss. It also advocates for incorporating palaeo‐ecological data into environmental assessments, ensuring that conservation strategies are informed by a comprehensive understanding of long‐term ecological changes. By bridging historical and contemporary data, decision‐makers can develop more effective restoration measures, safeguarding biodiversity and ecosystem services not only in New Zealand but also in other regions experiencing similar anthropogenic pressures. These integrative approaches are critical for restoring degraded habitats, achieving sustainable fisheries, and overcoming SBS on a global scale.

## Author Contributions

**Julian Lilkendey:** funding acquisition, conceptualization, data curation, investigation, formal analysis, methodology, supervision, validation, visualization, writing – original draft, writing – review and editing. **Jens Hegg:** data curation, formal analysis, methodology, resources, software, validation, visualization, writing – original draft. **Matthew Campbell:** data curation, formal analysis, funding acquisition, investigation, project administration, resources, validation, writing – review and editing. **Jingjing Zhang:** formal analysis, methodology, supervision, validation, writing – review and editing. **Harrison Raby:** formal analysis, investigation. **Malcolm Reid:** data curation, formal analysis, investigation, methodology, resources, writing – review and editing. **Monica Tromp:** investigation, resources. **Emma Ash:** investigation, resources. **Louise Furey:** investigation, resources. **Lindsey White:** funding acquisition, project administration, resources. **Richard Walter:** funding acquisition, project administration, writing – review and editing. **Armagan Sabetian:** funding acquisition, methodology, project administration, resources, supervision, validation, writing – review and editing.

## Conflicts of Interest

The authors declare no conflicts of interest.

## Supporting information


Appendix S1.

## Data Availability

The data that support the findings of this study are openly available in PANGAEA at https://doi.pangaea.de/10.1594/PANGAEA.971588. The model code for data analysis is available on GitHub at https://github.com/Jens‐Hegg/NZSnapperOtoDTW/ and Zenodo at https://doi.org/10.5281/zenodo.14567429.
