# Supplementary material for: Overcoming Shifting Baselines: Paleo‐Behaviour Reveals Industrial Revolution as Tipping Point
Source: Glob Chang Biol. 2025 Jan 25;31(1):e70038. doi: 10.1111/gcb.70038 (PMC11771676; doi:10.1111/gcb.70038)
Supplement: Supplementary file 1 — Appendix S1. [file GCB-31-e70038-s001.docx]

**Appendix**

**Slope Comparisons for Otolith Mass and Age by Assemblage**


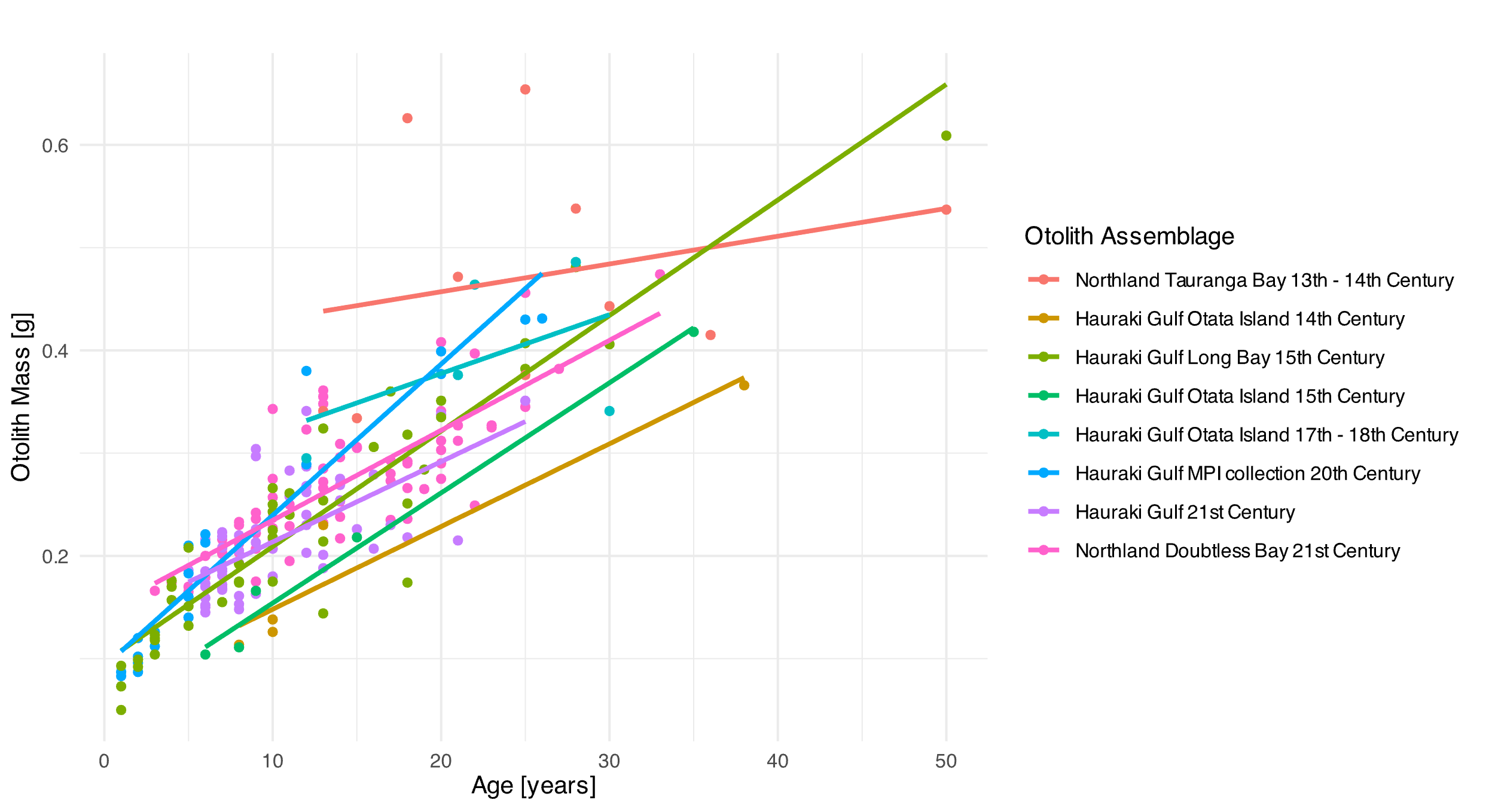


**Appendix Figure 1.** Pairwise comparisons of regression slopes for otolith mass versus age across epochs, highlighting significant differences and similarities in growth trends among archaeological, historical, and modern samples.

**Appendix Table 1.** Pairwise Comparisons of Slopes for the Otolith Mass-Age Relationship Across Otolith Assemblages. Differences in slope estimates between epochs are presented along with their standard errors (SE), degrees of freedom (df), t-ratios, and p-values. Significant contrasts (p < 0.05) highlight changes in the otolith growth dynamics across historical and modern snapper populations.

| **contrast** | **estimate** | **SE** | **df** | **t.ratio** | **p.value** |
| --- | --- | --- | --- | --- | --- |
| (Northland Tauranga Bay 13th - 14th Century) - Hauraki Gulf Otata Island 14th Century | 0.267613362 | 0.032034554 | 206 | 8.353896779 | 2.96E-13 |
| (Northland Tauranga Bay 13th - 14th Century) - Hauraki Gulf Long Bay 15th Century | 0.197994842 | 0.024835548 | 206 | 7.972235599 | 2.98E-12 |
| (Northland Tauranga Bay 13th - 14th Century) - Hauraki Gulf Otata Island 15th Century | 0.254336076 | 0.03173742 | 206 | 8.013760413 | 2.31E-12 |
| (Northland Tauranga Bay 13th - 14th Century) - (Hauraki Gulf Otata Island 17th - 18th Century) | 0.101659314 | 0.04518167 | 206 | 2.250012318 | 0.326704378 |
| (Northland Tauranga Bay 13th - 14th Century) - Hauraki Gulf MPI collection 20th Century | 0.158142414 | 0.02662797 | 206 | 5.938958623 | 3.35E-07 |
| (Northland Tauranga Bay 13th - 14th Century) - Hauraki Gulf 21st Century | 0.202671221 | 0.024826144 | 206 | 8.163620514 | 9.22E-13 |
| (Northland Tauranga Bay 13th - 14th Century) - Northland Doubtless Bay 21st Century | 0.179103482 | 0.02445211 | 206 | 7.324663585 | 1.49E-10 |
| Hauraki Gulf Otata Island 14th Century - Hauraki Gulf Long Bay 15th Century | -0.06961852 | 0.022521432 | 206 | -3.091211985 | 0.046065973 |
| Hauraki Gulf Otata Island 14th Century - Hauraki Gulf Otata Island 15th Century | -0.013277286 | 0.029961212 | 206 | -0.443149147 | 0.99984447 |
| Hauraki Gulf Otata Island 14th Century - (Hauraki Gulf Otata Island 17th - 18th Century) | -0.165954047 | 0.043952176 | 206 | -3.775786857 | 0.005056225 |
| Hauraki Gulf Otata Island 14th Century - Hauraki Gulf MPI collection 20th Century | -0.109470948 | 0.024483857 | 206 | -4.471148069 | 0.000338683 |
| Hauraki Gulf Otata Island 14th Century - Hauraki Gulf 21st Century | -0.06494214 | 0.022511061 | 206 | -2.884899113 | 0.080992885 |
| Hauraki Gulf Otata Island 14th Century - Northland Doubtless Bay 21st Century | -0.088509879 | 0.022097876 | 206 | -4.005356821 | 0.002171177 |
| Hauraki Gulf Long Bay 15th Century - Hauraki Gulf Otata Island 15th Century | 0.056341234 | 0.022096742 | 206 | 2.549753008 | 0.18093868 |
| Hauraki Gulf Long Bay 15th Century - (Hauraki Gulf Otata Island 17th - 18th Century) | -0.096335528 | 0.039017759 | 206 | -2.46901744 | 0.214785777 |
| Hauraki Gulf Long Bay 15th Century - Hauraki Gulf MPI collection 20th Century | -0.039852428 | 0.013785899 | 206 | -2.890811017 | 0.079750594 |
| Hauraki Gulf Long Bay 15th Century - Hauraki Gulf 21st Century | 0.004676379 | 0.009866086 | 206 | 0.473985275 | 0.999756103 |
| Hauraki Gulf Long Bay 15th Century - Northland Doubtless Bay 21st Century | -0.018891359 | 0.008883012 | 206 | -2.126683924 | 0.401372368 |
| Hauraki Gulf Otata Island 15th Century - (Hauraki Gulf Otata Island 17th - 18th Century) | -0.152676762 | 0.043736082 | 206 | -3.490865093 | 0.013457676 |
| Hauraki Gulf Otata Island 15th Century - Hauraki Gulf MPI collection 20th Century | -0.096193662 | 0.024093782 | 206 | -3.992468308 | 0.002279622 |
| Hauraki Gulf Otata Island 15th Century - Hauraki Gulf 21st Century | -0.051664855 | 0.022086172 | 206 | -2.33923991 | 0.277655639 |
| Hauraki Gulf Otata Island 15th Century - Northland Doubtless Bay 21st Century | -0.075232594 | 0.021664885 | 206 | -3.472559081 | 0.014291362 |
| (Hauraki Gulf Otata Island 17th - 18th Century) - Hauraki Gulf MPI collection 20th Century | 0.056483099 | 0.040182457 | 206 | 1.405665639 | 0.853858988 |
| (Hauraki Gulf Otata Island 17th - 18th Century) - Hauraki Gulf 21st Century | 0.101011907 | 0.039011774 | 206 | 2.589267205 | 0.165837811 |
| (Hauraki Gulf Otata Island 17th - 18th Century) - Northland Doubtless Bay 21st Century | 0.077444168 | 0.038774821 | 206 | 1.997279821 | 0.486248902 |
| Hauraki Gulf MPI collection 20th Century - Hauraki Gulf 21st Century | 0.044528808 | 0.01376895 | 206 | 3.234001619 | 0.030281575 |
| Hauraki Gulf MPI collection 20th Century - Northland Doubtless Bay 21st Century | 0.020961069 | 0.013082517 | 206 | 1.602219874 | 0.748554827 |
| Hauraki Gulf 21st Century - Northland Doubtless Bay 21st Century | -0.023567739 | 0.008856686 | 206 | -2.661010882 | 0.140817466 |
